# Measuring the Rise and Fall of Plasma 25-Hydroxyvitamin D Concentrations in Blue-Tongued Skinks (*Tiliqua scincoides*) Following Ultraviolet B Exposure and Withdrawal

**DOI:** 10.3390/vetsci12100965

**Published:** 2025-10-09

**Authors:** Ashleigh Godke, Haerin Rhim, M. Graciela Aguilar, Keishla Marrero-Acosta, Mark A. Mitchell

**Affiliations:** Department of Veterinary Clinical Sciences, School of Veterinary Medicine, Louisiana State University, Baton Rouge, LA 70803, USA; agodke3@lsu.edu (A.G.); mmitchell@lsu.edu (M.A.M.)

**Keywords:** reptile, skink, lizard, UVB, diet, vitamin D, deficiency, calcifediol, calcidiol

## Abstract

**Simple Summary:**

Blue-tongued skinks (*Tiliqua scincoides*) are popular pet reptiles, but there is limited evidence-based information about their care. For example, the method they use to obtain vitamin D—a nutrient vital to reptile health—is still unknown. This study investigated whether these skinks can meet their vitamin D needs through diet alone or require ultraviolet B (UVB) exposure. Skinks were raised on a wet cat food containing vitamin D, then exposed to UVB light for either 12 or 2 h/day for 4 weeks. The baseline vitamin D concentrations were low; however, UVB exposure dramatically increased these values. After the UVB lighting was removed, vitamin D concentrations decreased for the 12-hour and 2-hour groups, requiring more than 7 and 4 months for the values to return to baseline, respectively. These results indicate that blue-tongued skinks can utilize UVB light to synthesize vitamin D.

**Abstract:**

Species-specific husbandry guidelines remain limited for blue-tongued skinks (*Tiliqua scincoides*), especially in relation to ultraviolet B (UVB) lighting and vitamin D requirements. This study aimed to determine whether UVB exposure is necessary for these skinks and how long 25-hydroxyvitamin D (25-OHD) concentrations persist after UVB withdrawal. Eleven adult skinks who had been fed with wet cat food were exposed to 12 or 2 h of UVB per day for four weeks. Plasma 25-OHD concentrations were very low at the baseline, and significantly increased post-UVB in both groups (*p* < 0.01), with the 12-hour group increasing from baseline concentrations of 18.5 [12.8–20.5] nmol/L to 820 [730–1251.3] nmol/L and the 2-hour group increasing from baseline concentrations of 22 [15.5–22] nmol/L to 635 [401–892.5] nmol/L. Following the discontinuation of UVB exposure, 25-OHD gradually declined and was not significantly different from baseline concentrations at 7 and 4 months for the 12-hour and 2-hour groups, respectively. Dietary vitamin D_3_ (2.5 IU/g as dry matter basis), provided through wet cat food alone, appeared insufficient to support sustained plasma 25-OHD concentrations. These findings strongly suggest that blue-tongued skinks rely on UVB exposure to increase their 25-OHD concentrations. Moreover, the shorter 2-hour exposure provided a significant rise in 25-OHD concentrations and remained above baseline for 4 months, suggesting the shorter exposure can benefit these animals, while potentially reducing secondary risks associated with UVB exposure.

## 1. Introduction

Blue-tongued skinks (*Tiliqua scincoides*) have gained popularity in the pet reptile industry due to their docile temperament and ease of handling. However, despite their widespread presence in captivity, species-specific husbandry guidelines remain limited, particularly regarding ultraviolet B (UVB) exposure and vitamin D requirements. They are diurnal omnivores that are native to the northern and eastern regions of Australia and New Guinea [1]. Their ideal diet comprises approximately 50% vegetables, 25% fruits, and 25% invertebrates (mainly snails) [2]. In the wild, they predominantly bask in full sunlight with occasional partial sun exposure, typically experiencing a UV Index (UVI) ranging from 0.7 to 2.6, which corresponds to Ferguson Zones 2–3 [3,4]. Based on these data, blue-tongued skinks require moderate UVB exposure.

UVB radiation (290–315 nm) is essential for endogenous vitamin D synthesis in many reptile species. It initiates a photochemical reaction in the skin, transforming provitamin D_3_ (7-dehydrocholesterol) into previtamin D_3_, which is then thermally isomerized into vitamin D_3_ (cholecalciferol) [4,5]. Under prolonged UVB exposure, previtamin D_3_ is further converted into inactive photoproducts such as lumisterol and tachysterol, which help prevent the overproduction of vitamin D. In the liver, cholecalciferol is hydroxylated to 25-hydroxyvitamin D_3_ (25-OHD_3_; calcifediol, calcidiol), the major circulating form and the most reliable indicator of vitamin D status in blood. This is subsequently hydroxylated in the kidney to form 1,25-dihydroxyvitamin D_3_ (calcitriol), the biologically active form [6]. As a hormone, vitamin D plays a critical role in calcium homeostasis, cardiovascular function, immune regulation, and reproduction [7,8].

While animals may obtain vitamin D from two sources—dietary intake, UVB-induced synthesis [9]—basking is the primary pathway of acquisition for many reptiles because their natural diet, consisting mainly of invertebrates and plant matter, tends to contain low levels of vitamin D_3_ [5]. The relative dependence on each source varies considerably among vertebrate species [10]. Nonetheless, previous studies have demonstrated that most reptiles, regardless of their diet or whether they are diurnal, nocturnal, or crepuscular, experience an increase in serum 25-OHD_3_ concentrations following UVB exposure [11,12,13,14,15,16]. This supports the conclusion that the photoconversion pathway plays a crucial role in a wide range of reptile species [17]. Furthermore, within a single species, self-regulation based on physiological needs has been confirmed in panther chameleons (*Furcifer pardalis*) through their behavioral adjustment of UVB exposure according to the amount of vitamin D consumed in their diet [12].

Most of these reptile studies used standard 12-hour UVB photoperiods for diurnal species. However, not all reptiles naturally bask for extended periods, and overexposure to UVB carries documented risks, including DNA damage in the epidermis and corneal epithelium degradation [18]. Recent research on leopard geckos (*Eublepharis macularius*)—a crepuscular species—has shown that even brief UVB exposure for 120 min can significantly raise 25-OHD_3_ concentrations [15]. To address these concerns and investigate how different exposure durations impact vitamin D concentrations, we established two distinct UVB photoperiods in this study.

This study aimed to investigate the rise and fall of plasma 25-OHD_3_ concentrations in blue-tongued skinks that were fed a wet cat food formulated to meet feline vitamin D requirements in response to two different UVB exposure conditions (12- and 2-hour daily) for four weeks. Additionally, we monitored the rate of decline in 25-OHD_3_ concentrations following the cessation of UVB exposure to assess how these animals process this hormone. Our hypotheses were as follows: (1) Skinks will exhibit 25-OHD concentrations > 50 nmol/L when supplemented from cat food prior to UVB exposure; (2) Skinks in both the 12-hour and 2-hour UVB exposure groups will exhibit significantly increased 25-OHD_3_ concentrations compared to their own respective baseline concentrations; and (3) 25-OHD_3_ concentrations will decrease over time when UVB is removed.

## 2. Materials and Methods

A prospective, randomized experimental study was conducted under the regulations set forth by the Louisiana State University Institutional Animal Care and Use Committee (22-050).

### 2.1. Materials

Eleven adult (1.5 years, 694 ± 63.8 g) non-sexed northern blue-tongued skinks were used in the study. All individuals were clinically healthy based on physical examinations performed prior to the study; however, a total of 7 out of 11 skinks did show evidence of kinked tails. We observed no meaningful variation in either baseline or post-UVB 25-OHD concentrations when comparing the two groups, suggesting that this physical characteristic did not confound the results. The skinks were individually housed in 91 × 45 × 33 cm (36 × 18 × 13”) enclosures (Rubbermaid, Wooster, OH, USA) in a room with ambient temperature and humidity of 30 °C (86 °F) and 35%, respectively. Each enclosure lid had a rectangular mesh window that was big enough to allow ventilation and light delivery efficiently. The skinks were exposed to a 12-hour photoperiod using ambient fluorescent lighting; no UVB lighting was provided before the study. A non-light-penetrating black plastic shelter (31 × 20 × 8 cm [12 × 8 × 3”]) was placed on one side of the enclosure that allowed the skinks to fully conceal themselves. Fresh chlorinated tap water was provided daily, and the skinks were fed 20 g of wet cat food (Wellness, Burlington, MA, USA) three times a week. This specific cat food is what the skinks were fed at the breeder, and we maintained a single-source diet for the skinks to reduce any variability in vitamin D concentrations based on skink dietary preferences.

UVB lighting was supplied using a 26-watt coil bulb (Sun Glow 5.0 UVB; Fluker’s, Port Allen, LA, USA) mounted in a reflective dome (Sun Dome; Fluker’s) and placed on top of each enclosure lid. Irradiance levels were set to 1.5–2.0 UVI at the substrate level in the center of each enclosure and measured using two UVB meters (SOLARMETER Model 6.2R and 6.5R; Glenside, PA, USA). UVB output was monitored weekly at the center and sides of each enclosure to ensure consistency throughout the trials. To prevent indirect exposure between groups and individuals, each cage was isolated by blocking it with light-impermeable cardboard.

### 2.2. Study Designs

Blood samples were collected from the ventral tail vein of all eleven skinks using a 25-gauge needle fastened to a 1 mL syringe at the baseline and every four weeks, depending on the trials. Samples were placed in a lithium heparin tube (BD Microtainer^®^; Becton, Dickinson and Company, Franklin Lakes, NJ, USA). Plasma was then separated by centrifugation at 4000 rpm for 5 min and stored in cryovials (VWR^®^, Wayne, PA, USA). Samples were processed within 15–60 min of sample collection. All sampling was performed at the same time each morning, prior to feeding, to minimize potential circadian effects on 25-OHD_3_ concentrations. Plasma samples were frozen at −80 °C and transported on frozen gel packs to the Veterinary Diagnostic Laboratory at Michigan State University (MSU; East Lansing, MI, USA). All samples were processed within 30 days of collection. Plasma 25-OHD concentrations were quantified using a direct competitive chemiluminescent assay (Liaison^®^ XL; DiaSorin Inc., Stillwater, MN, USA) by the clinical laboratory [19]. This test has 100% cross-activity between 25-OHD_2_ and 25-OHD_3_; however, the increased 25-OHD concentrations after UVB exposure were likely to be contributed by 25-OHD_3_.

#### 2.2.1. Phase I (12- and 2-Hour of UVB Exposure)

Six skinks were randomly selected using a random number generator (random.org) to receive UVB exposure for 12 h per day (8:00 a.m. to 8:00 p.m.) for four weeks (28 days). The remaining five skinks served as the control group and were not exposed to UVB during this phase. Blood samples were collected at baseline and the end of the 4-week period. Following this, the UVB lights for the treatment group were turned off to begin the decay phase.

Subsequently, the five skinks previously used as controls were assigned to a new treatment group and received UVB exposure for 2 h daily (8:00 a.m. to 10:00 a.m.) for four weeks. Blood samples were collected at the end of the 4-week exposure period, and the UVB lighting was discontinued after the samples were collected. These skinks served as their own control.

#### 2.2.2. Phase II (Decline Following Cessation of UVB Exposure)

After a 4-week UVB exposure period, 25-OHD concentrations were generally measured at monthly (4-week) intervals following the cessation of UVB lighting. Post-UVB samples were collected at 2, 3, 6, 7, 8, and 9 months from cessation in the 12-hour group, and at 3, 4, 5, and 6 months in the 2-hour group. Months 4 and 5 for the 12-hour group and month 2 for the 2-hour group could not be collected in a timely manner for logistical reasons and were therefore skipped.

### 2.3. Statistical Analysis

The sample size for the 12-hour UVB study was determined using the following a priori data: an alpha = 0.05, power = 0.8, an expected difference in mean 25-OHD concentrations between the control and the treatment groups of 25 nmol/L, and standard deviations between groups of 12 nmol/L. The sample size for the 2-hour study was determined using a paired samples comparison with an alpha = 0.05, a power = 0.9, an expected difference between the baseline and week 4 samples of 25 nmol/L, and a standard deviation for each group of 12 nmol/L. A higher a priori power was used for the 2-hour study because it was only a within-subject study design.

The distribution of the 25-OHD data and the residuals for the data were assessed using the Shapiro–Wilk test, Q-Q plots, skewness, kurtosis, and histograms. Normally distributed continuous data were presented by the mean ± standard deviation (SD), while non-normally distributed data were shown as median [interquartile range (IQR)] with minimum-maximum values in parentheses.

Weekly UVB output was assessed using repeated-measures ANOVA with time as the within-subject factor and group (12- and 2-hour groups) as the between-subject factor. Where significant interaction effects were observed, follow-up repeated-measures ANOVA with Bonferroni adjustment was conducted within each group to evaluate time-specific changes.

Although the 4-week data were not normally distributed, the residuals met the assumption of normality. Therefore, repeated-measures ANOVA was used to analyze changes in 25-OHD concentrations during the 4-week UVB exposures (Phase I), with time (day 0 and day 28) as the within-subject factor and group (control and 12-hour group) as the between-subject factor. Paired samples t-tests were subsequently performed post hoc to examine time effects within each group. For the 2-hour exposure group, a paired samples t-test was used to measure differences in 25-OHD concentrations between days 0 and 28; there was no between-subject variable for this trial. While parametric tests were applied based on the normality of residuals, all descriptive statistics are presented as medians [IQR] because the partial raw data were not normally distributed.

Since partial post-UVB (Phase II) data were not normally distributed, a Friedman test was utilized to analyze the temporal decrease in 25-OHD concentrations following cessation of UVB exposure. If the model was significant, post hoc pairwise comparisons were performed using the Wilcoxon signed-rank test.

All data were analyzed using SPSS V26.0 (IBM Statistics, Armonk, NY, USA). A *p*-value < 0.05 was used to determine statistical significance. Additionally, the half-life of 25-OHD was estimated using an exponential decay model in Microsoft Excel V2016 (Microsoft, Redmond, WA, USA).

## 3. Results

The average UVB irradiance at the center of the enclosures was 48.25 ± 5.2 (36–58) µW/cm^2^. Repeated measures ANOVA revealed no significant effect of week (*F*[2,19] = 2.053, *p* = 0.153) or group (*F*[1,9] = 0.286, *p* = 0.606), but a significant week × group interaction was detected (*F*[2,19] = 4.75, *p* = 0.02). Follow-up repeated measures ANOVA performed within each group showed a significant change over time only in the 2-hour group (*F*[2,7] = 7.892, *p* = 0.015), specifically between weeks 2 (52 ± 3.4 µW/cm^2^) and 4 (47 ± 4.6 µW/cm^2^; *p* = 0.037). However, this difference was attributed to an unusually high UVB output during week 2, rather than a consistent decline over time, and was therefore interpreted as incidental.

### 3.1. Phase I (12- and 2-Hour of UVB Exposure)

Plasma 25-OHD concentrations are presented in Table 1. Baseline concentrations in all skinks, reflecting intake from wet cat food alone, were uniformly low (16.73 ± 3.7 [9–22] nmol/L).

In the 12-hour exposure trial, there were significant main effects for group (*F*[1,9] = 24.1, *p* = 0.001), time (*F*[1,9] = 25.1, *p* = 0.001), and group × time (*F*[1,9] = 24.8, *p* = 0.001). While the control group showed no significant change over time (*t*[4] = −1.56, *p* = 0.2), the 12-hour UVB-exposed group exhibited marked increases in 25-OHD concentration after 4 weeks (*t*[5] = −5.517, *p* = 0.003), with an approximate 44-fold increase from 18.5 [12.8–20.5] to 820 [730–1251.3] nmol/L. The 2-hour group also demonstrated a significant difference (*t*[4] = −4.94, *p* = 0.008), with a 29-fold increase from 22 [15.5–22] to 635 [401–892.5] nmol/L.

### 3.2. Phase II (Decline Following Cessation of UVB Exposure)

A significant decline in 25-OHD concentration over time was observed in both the 12-hour group (*χ*^2^[6] = 35.34, *p* < 0.001) and 2-hour group (*χ*^2^[4] = 19.36, *p* < 0.001), as shown in Table 2.

Although certain time points were missing in both groups, their 25-OHD concentrations significantly differed until post-7 months in the 12-hour group and post-4 months in the 2-hour group. Their concentrations gradually decreased and returned to near-baseline concentrations post 9 months in the 12-hour group (27.5 [21.5–37] nmol/L) and post 6 months in the 2-hour group (27 [22–31.5] nmol/L). The estimated half-life of 25-OHD_3_ ranged from 32 to 77 days, with an average of 59.8 days in the 12-hour group and 38.5 days in the 2-hour group.

## 4. Discussion

Although cat-based diets are often used among blue-tongued skink caretakers, our study unexpectedly revealed that their 25-OHD concentrations were very low (<22 nmol/L), based on communication with MSU from 89 previous submissions in this species. We initially assumed that the cat diet would provide sufficient vitamin D, as cats rely on dietary intake rather than endogenous synthesis [10]. However, this low concentration suggests that the skinks likely did not obtain sufficient vitamin D solely from the wet cat food, especially considering the sharp rise in their calcifediol concentrations after UVB exposure. Supporting this, 7 of 11 skinks exhibited signs of kinked tails prior to the study, likely resulting from nutritional secondary hyperparathyroidism, also known as metabolic bone disease. This condition commonly derives from inadequate calcium absorption due to hypovitaminosis D, which is one of the most common problems found in captive reptiles [20]. Human studies have revealed that calcium absorption is significantly impaired (10–15%) when vitamin D is absent, compared to a state where adequate vitamin D is present (30–40%) [21]. Two possible reasons for these low baseline 25-OHD concentrations were: (1) the cat food did not contain enough vitamin D_3_ to meet blue-tongued skinks’ requirements, or (2) the skinks do not effectively utilize the dietary vitamin D_3_.

The vitamin D_3_ in the wet cat food we provided (0.6 IU/g as fed basis [AF], 2.5 IU/g as dry matter basis [DM]) exceeded the nutritional requirements for cats, which depend solely on dietary vitamin D uptake. Although the skinks did not always consume the entire portion, with a total of 60 g offered weekly, their approximate vitamin D_3_ intake was 5 IU/day AF or about 7 IU/kg/day AF when divided by body weight. While species-specific nutritional requirements are lacking in most reptile species, this intake was considered appropriate but on the lower end of the range because the recommended dietary range for omnivorous reptiles is 0.5–2.5 IU/g AF [3,22]. However, this recommendation does not imply that dietary vitamin D alone is sufficient. For instance, green iguanas (*Iguana iguana*) fed a diet containing 3 IU/g vitamin D_3_ DM and bearded dragons (*Pogona vitticeps*) receiving approximately 1–3.8 IU/g AF still showed low circulating 25-OHD_3_ concentrations without UV exposure [23,24,25]. Similarly, neither a dietary vitamin D_3_ concentration of 0.45 IU/g AF nor whole rodent prey increased 25-OHD_3_ concentrations in Komodo dragons (*Varanus komodoensis*) without UVB exposure, despite these being carnivorous lizards that were expected to obtain most of their requirements through their diet [26,27]. Likewise, given that blue-tongued skinks naturally consume very low amounts of vitamin D_3_ from their omnivorous diet, they are likely to be heavily dependent on UVB exposure for their vitamin D fulfillment.

In contrast, a study conducted in 3- to 4-week-old juvenile green iguanas demonstrated that vitamin D supplements (1.245 IU/g AF) led to significantly better growth rates compared to individuals that did not receive supplements, despite both groups being kept without UVB [28]. Moreover, a single dose of 10 IU/g body weight vitamin D_2_ and D_3_-combined oil suspension successfully increased blood vitamin D concentrations in green iguanas, far exceeding the daily intake provided by diets containing 2.5 IU/g dry matter as used in our study [24]. Similarly, a six-fold increase above baseline was reported in black-throated monitor lizards (*Varanus albigularis*) when 5–15 IU/g D_3_ was fed once weekly for 92 days [29]. These studies suggest that further research is required to determine whether blue-tongued skinks can utilize oral vitamin D_3_ if provided at higher doses or in alternative formulations. Although the exact threshold for vitamin D toxicity in reptiles is not yet clear, a tentative maximum tolerance of 5 IU/g is generally considered for many species [30].

Another possible hypothesis is that the skinks were able to absorb dietary vitamin D_3_, but its utilization was impaired due to excessive vitamin A present in the cat food (327 IU/g DM). Recommendations of vitamin A for omnivorous reptiles range 3–9 IU/g DM, preferably in the form of beta-carotene from natural food sources [31,32]. Overfeeding vitamin A at levels exceeding 100 times the recommended amount has been associated with a high risk of hypervitaminosis A [30]. Since vitamin A has been shown to induce hypovitaminosis D when present in excessive amounts, it could also have contributed to the poor vitamin D status in our skinks, as it has been described in veiled chameleons (*Chamaeleo calyptratus*) [33,34,35]. Additional studies are needed to clarify whether lower dietary vitamin A would enhance the absorption and utilization of dietary vitamin D_3_ in blue-tongued skinks. This highlights the broader need for comprehensive research investigating the impact of both natural diets and vitamin D-supplemented diets on blood vitamin D levels.

Once they were exposed to daily UVB irradiation (48.25 ± 5.2 µW/cm^2^), their 25-OHD_3_ concentrations surged dramatically, regardless of exposure duration—showing an 18- to 76-fold increase from baseline in the 2-hour group, and 36- to 94-fold increase in the 12-hour group. Moreover, the upper range of post-exposure concentrations in this study is comparable to the highest concentrations of 25-OHD_3_ ever reported in reptiles following UVB exposure [7,10,11,12,13,14,15,16,25]. Our results align with previous studies in various herbivorous and omnivorous reptiles, which consistently demonstrate that UVB exposure is the primary driver of vitamin D synthesis. Although a time-dependent effect was initially anticipated, even the 2-hour daily exposure resulted in a substantial rise in 25-OHD_3_ concentrations, suggesting that short exposure may suffice for blue-tongued skinks. Additionally, the actual difference in UVB exposure between the two groups may have been smaller than the scheduled durations imply, as evidenced by overlapping 25-OHD_3_ concentrations. Notably, this effect occurred despite the relatively low irradiance—comparable to levels found in shaded natural environments—and the availability of a light-impermeable shelter that allowed voluntary hiding, which may have partially contributed to the wide variation observed within the group. Although information on the sunlight exposure patterns of blue-tongued skinks in the wild is limited, this finding demonstrates that a 12-hour photoperiod is likely excessive, even considering they have basking periods where UVI might reach 2–3. This shorter period can be a more efficient and potentially safer husbandry practice, reducing energy costs and the risk of overexposure.

Unfortunately, no evidence-based data currently exist on the required vitamin D quantity or the physiological reference range of 25-OHD_3_ in wild populations of this species. According to the MSU’s diagnostic laboratory, the suggested range for blue-tongued skinks was between 103 and 545 nmol/L based on submitted samples. While physiological differences exist across species, studies in wild or near-wild populations of various squamates have shown typical ranges around 100–400 nmol/L [11,36]. Some species exhibit even broader variation; wild-captured Galapagos land iguanas (*Conolophus* spp.) showed values from 43 to 890 nmol/L [37], and wild Ricord’s iguanas (*Cyclura ricordii*) had concentrations ranging from 250 to 1118 nmol/L [38]. The authors of the iguana studies suggested that these animals are adapted to manage sun exposure in ways that maintain high 25-OHD_3_ concentrations without resulting in toxicity, and that seasonal or thermal fluctuations may account for the observed variation. Reptiles are believed to detect UVB and regulate basking based upon their needs, despite not perceiving UVB; they can visually perceive UVA [39,40]. A finding that eastern fence lizards (*Sceloporus undulatus*) opt for UV exposure even when it means tolerating body temperatures above their preferred range also supports this [41]. However, since a constant ambient temperature was provided during the study, preventing the skinks from engaging in the thermal trade-off behavior that might be observed in a more complex natural environment. This self-regulation has also been demonstrated in chameleons adjusting their basking based on dietary vitamin D intake, and vice versa in bearded dragons [5,12,25]. Given that similarly elevated concentrations are seen in blue-tongued skinks under UVB supplementation, additional investigation on wild populations is crucial to establish a physiological reference range and optimize husbandry practices.

Vitamin D toxicity has been described as resulting from excessive oral intake because excessive UVB-derived previtamin D_3_ is mostly converted to inert metabolites under prolonged exposure and stored [21,42]. It is generally understood that extended UV exposure does not lead to a continuous increase in circulating 25-OHD_3_ concentrations [10]. Although the precise toxicity threshold remains unclear, in humans, concentrations above 374 nmol/L or a calcium × phosphorus product greater than 60 mg^2^/dl^2^ have been considered risk factors for soft tissue mineralization [21,43]. Nevertheless, several reptile species appear to tolerate relatively high vitamin D concentrations acquired via UVB, as described earlier. Whether chronic deficiency can contribute to extreme post-exposure surges remains unknown. However, vitamin D-depleted green iguanas exhibited a similar surge, reaching 952 nmol/L after being provided with a UVB lamp [44].

One case report described vitamin D toxicosis in a blue-tongued skink that had received a high vitamin D supplement along with UVB exposure [45]. In that case, the elevated 25-OHD_3_ concentration (768 nmol/L) and multifocal calcifications were attributed to the daily vitamin and calcium supplements. Interestingly, the 25-OHD_3_ concentration fell within the high range we observed in our post-UVB results. This raises the question of whether the elevated value was primarily due to dietary intake, or whether blue-tongued skinks may have a limited tolerance for dietary vitamin D_3_ beyond a certain threshold. However, our skinks in this study were under 2 years old, which may have masked underlying conditions—especially when considering that the affected individual in the case report was 10 years old. The absence of radiological or histopathological evaluations in our colonies also limited a more comprehensive assessment of their health status; however, the skinks in this colony continue to thrive 18 months post-this study. Fatal metastatic mineralization due to supplement overdose (10–20 IU/g AF) has also been reported in spot-tailed earless lizards (*Holbrookia* spp.) [46]. Notably, soft tissue mineralization was observed only in panther chameleons receiving high dietary vitamin A, regardless of their vitamin D concentrations [47], highlighting the need for broader investigation into interactions of these nutrients.

When UVB was discontinued, there was a significant decline in the 25-OHD_3_ concentrations over time. Plasma concentrations waned by 68% two months after discontinuation and by 83% after 3 months. This gradual reduction suggests that, in the absence of UVB exposure, blue-tongued skinks progressively deplete their stored vitamin D_3_—even when provided 2.5 IU/g DM of dietary vitamin D_3_. This relatively prolonged half-life, ranging from one to two months, may be due to the strong binding affinity of 25-OHD_3_ to vitamin D-binding protein (DBP), whereas dietary vitamin D_3_ (cholecalciferol) binds with lower affinity [48]. Furthermore, photochemical metabolites generated in abundance during cutaneous synthesis can be converted back into previtamin D_3_ via reverse reactions [12], although the exact duration over which these metabolites can be stored remains unclear. These mechanisms may help explain why skinks in the 12-hour group retained 25-OHD_3_ longer than those in the 2-hour exposure group. In black-throated monitors, 25-OHD_3_ decreased at a relatively constant rate during the deprivation period, with a half-life of 128–139 days when fed vitamin D-free crickets [29]. This implies the physiological ability of these animals to maintain their vitamin requirements in the wild without needing daily, prolonged sun exposure. These findings collectively indicate that, once elevated, 25-OHD_3_ levels can be maintained for a substantial period. Therefore, intermittent UVB exposure may be sufficient to meet vitamin D requirements in some squamate species. Furthermore, the fact that a 2-hour exposure sustained high concentrations for four months suggests that a specific dosing regimen can be established for this species. This approach has been successfully demonstrated in Komodo dragons, where periodic sun exposure sustained elevated 25-OHD_3_ concentrations throughout non-exposure periods [27]. Future studies are warranted to determine the optimal frequency and duration necessary for maintaining adequate vitamin D status in blue-tongued skinks. Such minimal yet effective exposure may reduce the risk of UVB-related complications, as seen in a blue-tongued skink diagnosed with photokeratitis and photodermatitis [18].

## 5. Conclusions

Our findings confirmed that UVB exposure is likely the primary driver of vitamin D_3_ synthesis in blue-tongued skinks, with even short daily exposure leading to substantial increases in plasma 25-OHD_3_ concentrations. Dietary vitamin D_3_ from wet cat food alone was insufficient to raise concentrations without UVB, and elevated concentrations were maintained for weeks after UVB was discontinued. Future research on wild skinks will elucidate optimized approaches to husbandry practices.

## Figures and Tables

**Table 1:** Plasma 25-hydroxyvitamin D concentrations (nmol/L) in blue-tongued skinks before and after being exposed to 12 or 2 h of daily UVB over four weeks. Median and interquartile range (IQR) and minimum-maximum values (Min-Max) were reported. *p* values were obtained from paired t-tests within each group.

Group	Time	Median	IQR	Min–Max	*p* Value
12-hour UVB					
Control	Day 0	16	14–19	14–21	
	Day 28	22	15.5–22	14–22	0.194
Treatment	Day 0	18.5	12.8–20.5	9–22	
	Day 28	820	730–1251.3	730–1870	0.003 *
2-hour UVB					
Treatment	Day 0	22	15.5–22	14–22	
	Day 28	635	401–892.5	297–1070	0.008 *

* *p* < 0.05.

**Table 2:** Plasma 25-hydroxyvitamin D (nmol/L) concentrations in blue-tongued skinks over time following cessation of four-week UVB exposure. Median and interquartile range (IQR) with minimum-maximum values (Min–Max) were reported. *p* values were calculated from the pairwise comparisons performed relative to the baseline.

UVB	Time	Median	IQR	Min–Max	*p* Value
12-hour	Baseline	18.5	12.8–20.5	9–22	
	Week 4 *	820	730–1251.3	730–1870	<0.001
	Post 2M *	271.5	189–429.3	159–640	<0.001
	Post 3M *	129	92.8–230.3	59–447	0.002
	Post 7M *	44.5	33–60.3	21–67	0.038
	Post 8M	43.5	29.5–55	25–64	0.071
	Post 9M	27.5	21.5–37	22–34	0.504
2-hour	Baseline	22	15.5–22	14–22	
	Week 4 *	635	401–892.5	297–1070	<0.001
	Post 4M *	46	35–52.5	31–56	0.003
	Post 5M	30	27–42	26–49	0.072
	Post 6M	27	22–31.5	20–46	0.230

* *p* < 0.05.

## Data Availability

The original datasets presented in the study are included in the article, and further inquiries can be directed to the corresponding author.
